# Examining Heterogeneity of Food Fortification and Biofortification Business Models: Emerging Evidence for a Typology

**DOI:** 10.3390/nu13041233

**Published:** 2021-04-08

**Authors:** Baqir Lalani, Rania Hassan, Ben Bennett

**Affiliations:** Natural Resources Institute, Medway Campus, University of Greenwich, Central Avenue, Chatham Maritime, Kent ME4 4TB, UK; R.Hassan@greenwich.ac.uk (R.H.); ben.bennett@greenwich.ac.uk (B.B.)

**Keywords:** fortification, biofortification, business models, typologies, nonmetric dimensional scaling (NMDS)

## Abstract

Efforts to address Micronutrient deficiencies (MNDs) in lower-and middle-income countries (LMICs) have been gaining pace in recent years. Commodities such as staple foods (e.g., cereals, roots, and tubers) and condiments (e.g., salt) have been targeted as ‘vehicles’ for fortification and biofortification through numerous projects and initiatives. To date, there have been mixed experiences with delivery and coverage with very little documented on the range of business models applied in different geographies, business conditions and polities and this makes classification and measurement of success and failure difficult. This research aims to address this gap in knowledge through proposing a typology that clarifies similarities (internal heterogeneity) and differences (external heterogeneity) between models and that can allow all types to be defined by the combination of attributes. Building on a comprehensive literature review; NVivo was used to code initiatives from 34 key references (955 cases in total) which have been grouped into 17 categories. Using non-metric multidimensional scaling (NMDS) we find evidence of four business model groupings that typify fortification initiatives: (1) Large-scale private, unregulated, (2) Mixed-Scale, private, unregulated (3) Large-scale, public-private, regulated; and (4) Large-scale, private, regulated. We characterise these four groups with country examples and suggest that this typology can help the discourse around viability of food fortification initiatives.

## 1. Introduction

Efforts to address micronutrient deficiency (MND) in lower-and middle-income countries (LMICs) are gaining pace [1]. One avenue has been through the fortification of staple foods (e.g., cereals, roots, and tubers) and condiments (e.g., salt) which have been targeted as ‘vehicles’ for fortification and biofortification through numerous projects and initiatives e.g., [2,3]. However, while some initiatives seem viable and successful, many do not survive the initial project support, often provided by a donor. It is posited that the business model adopted for implementation of food fortification in each case may play a role in driving sustainability. This is crucial to future uptake and success of food fortification initiatives because of the importance of viability (financial, technical, social) to achieving the desired public health impacts. This issue of viability is, to date, is largely absent from the literature [4] and evidently valued by practitioners [5]. Furthermore, [6] explains at a higher level the potential impact of food fortification on public health (e.g., please refer to the ‘Impact model’ developed by [6]).

A comprehensive literature review (published in this journal) of business model success and failure has suggested a set of contexts and drivers that might guide future interventions [4]. Typically, food fortification is defined in three modes: point of cultivation (biofortification), point of consumption (home fortification) or at the point of food processing (industrial fortification) as the means to deliver the benefits [7]. WHO (2006) [8] define food fortification as ‘mass fortification’ (adding micronutrients to staples—often mandatory and government led), ‘targeted fortification’ (aimed foods at a particular subgroup of a population) and ‘market-driven’ (businesses adding micronutrients to staple foods for commercial gain). Other forms of food fortification include household and community fortification (often called ‘complementary food supplementation’), and biofortification (The European Commission uses the term ‘nutrient enriched crops’ rather than biofortification. Nutrient enriched crops exclude genetically modified organisms.) (the breeding or genetic modification of plants to improve their micronutrient content. The review covered these three forms of fortification: industrial fortification (mass fortification e.g., oil, salt), biofortification of staple foods (e.g., cereals, roots and tubers), and fortified complementary foods (In this category we also include Micronutrient powders (MNPs) used with complementary foods either through home fortification and/or point-of-use fortification e.g., school meals.) (targeted fortification). Contexts included: viability from the perspective of the core business, the food product being made, within the competitive framework of the business context and the societal context. Drivers of success and failure include the level of maturity of the business, the degree to which business targets are set and driven internally (by the business) or externally (by the government or donor), the scale of business and way that it coordinates itself at different scales, the setting and regulation of quality standards, the degree to which there is dependency on imported elements for production and the relative interplay between public and private sector participation in the model. We adopt five levels of business scale from [9] to help classify the different dynamics of business growth.

Level 1 refers to cooperation along value chains with minimum involvement from the private sector towards sustainable development—including nutrition. Level 2 relates to project-level partnerships such as linkage with investors, governments and research centres, whilst level 3 is more organised with industry-level alliances and a stronger commitment/organised approach to sustainable development goals. Level 4 consists of multi-stakeholder institution platforms and networks which can be formalised, or informal platforms, and consist of a high commitment to sustainable goals and development. Finally, level 5 consists of coordination between all the different levels some can be led by business, government or civil society [9]. 

A classification of business models for delivering fortified foods would facilitate broader empirical analysis of the range of models applied in different geographies, business conditions and polities. Types are common practice in qualitative social science and owe much to the original conceptualisation of Weber [10]. The concept presupposes that processes have attributes that have clear similarities (internal heterogeneity) and differences (external heterogeneity) and that this can allow all types to be defined by the combination of attributes. This allows reduction/concentration of complexity to a limited number of types which have relevance. More recently, efforts to systematise typologies have led to measurement of relationships to develop typologies, for example using grounded theory based on coding [11].

A primary aim of this study is to develop and characterise the business models and business parameters that drive successful food fortification and to propose a typology/typologies. This is important because food fortification is not cost neutral. Understanding the viability (financial, technical and social) of different approaches and balance of public and private commitment to food fortification as a means to achieve national and global public health goals can help garner support from these sectors and consumers for widespread implementation and uptake.

An explanation of the methodology is found in Section 2. Section 3 presents the results and proposed typology. Section 4 examines the different typologies with country examples. Section 5 provides concluding comments and discusses the implications for the design of fortification initiatives in LMICs. Our assumption is that we can answer questions about the reported drivers of success and failure in different food fortification business models, by analysing the relationships between ontology in the literature.

## 2. Materials and Methods

Kluge [12] distinguishes four stages of analysis for type construction: (1) development of relevant analysis dimensions; (2) grouping cases and analysis of empirical regularities; (3) analysis of meaningful relationships and type construction; and, (4) characterisation of constructed types. Considering the universe of business model types as outlined in Lalani et al. [4] as our starting point, and adopting the five stage analysis approach our initial relevant dimensions are shown in Table 1.

A combination of three software packages was used for this study: NVivo, Excel and R programming. Each software served a specific purpose in the data extraction and analysis process. For instance, NVivo was used to identify, code and group information from the literature [13]. Both Excel and R programming were used to explore and analyse data to construct the typology.

***Step 1: Literature search.*** Using articles identified in Lalani et al. [4], 34 articles were selected for this study (see Table A1). 

***Step 2: Identifying cases.*** NVivo was used as a word processing software to identify and code cases found in the 34 articles (full list in Table A1). Cases were selected based on two criteria, (1) type of fortification, and (2) type of fortificant or/and country. Information was then coded and grouped into 17 different dimensions seen in Table 2. 

***Step 3: Building the database.*** Cases identified in NVivo were then logged and organised into an Excel spreadsheet. Rows were used to represent one type of fortificant and one method of fortification. If multiple fortification methods/technologies were used, each were presented as a unique case. For example, Bibomix a micronutrient powder was entered into 14 different rows as it used 14 types of fortificants and one fortifying process. In total, 955 cases emerged from 79 countries, of which 36 types of food vehicles were used, with minerals accounting for 34% and vitamins for 28% (see Table 3). 

***Step 4: Statistical Analysis.*** Cases were narrowed from 955 to 255 to show programmes that either scored ‘successful’ or ‘unsuccessful’ and where country/region of operation had been identified. From this list, biofortification accounted for 33%, complementary foods 5% and fortification 62%. In total, 49 countries were identified, of which 76% were predominantly from lower-income countries. Functions such as VLOOKUP, PivotTables and Filers were particularly useful in exploring the data. A total of 23 articles were found relevant (see Table A1)

To ensure no duplication of data, we restructured the list of cases to make food vehicles the subject instead of fortificant type. Therefore, fortificants used to produce one product were grouped and presented as one unique case (i.e., Bibomix is now presented as one case instead of 14). This helped narrow the list from 255 to 103, see Section 3.1. for the full results. 

***Step 5: developing the typology and further analysis.*** To qualify the data and develop the typology, a scoring matrix was developed using five final index/indicators found in the master spreadsheet (see Table 4). A total of four criteria emerged: Commercial Viability (*CommVia*), Supportive Regulations/Legislation (*SuppReg*), Quality and Standards (*QltStnd*) and Target. Furthermore, to provide a macro assessment of each country type, a fifth criteria was developed using data from the Index of Economic Freedom [14] and named Governance environment (*GovEnv*). Each criteria weighed between 0 to 1 except for *SuppReg,* which ranged between 0 to 1.5 but was scaled down to 0 to 1 for the analysis (see Table 4). R software was used to develop a non-metric multidimensional scaling (NMDS) model [15,16] to form the typology.

Dendrograms show the hierarchical relationship between cases and are useful in revealing clusters [17,18,19]. NMDS produces output resembling a principal component analysis, but is robust in the face of non-linear measurement scales [20], and deviations from normality, and correlations between the measures used. An iterative method is used to find stable solutions. NMDS is particularly useful in mapping cases [21] and has been applied to other micronutrient studies such as [22,23].

### Limitations

A limitation of the study is combining the fortification and biofortification cases (typology exploration) given each operate under different circumstances (e.g., government regulation) and can be marketed to differing target markets. Another limitation relates to the perceptions of success which might be biased towards reporting on success rather than failures and/or involve those that are involved in these projects and therefore more likely to report success. It should be noted that this does not necessarily mean they were not successful just that success was not stated. In a few cases, some countries can also appear in two categories depending on the case-thus the specific country groupings should be treated with caution and evaluated on a case-by-case basis. 

## 3. Results

This section is therefore divided into three sections: In Section 3. 1, we first explored the cases with respect to their country groupings by income, fortification type and business model. In Section 3.2, we look at the success of programmes by country grouping using the four criteria developed to explore a typology (i.e., *CommVia*, *GovEnv*, *QltStnd, SuppReg* and *Target* see Table 4). The final Section 3.3, investigates whether groupings/typology can be established based on the categories identified.

### 3.1. Fortification Type, Business Model and Country Groupings

***Fortification and country type.*** Biofortification programmes accounted for 35% of the sample data, complementary foods 3% and fortification at 62% (full list of cases shown in Table A2). The largest group of countries in this study are from LMICs at 46%, followed by LIC (low-income countries) at 35%, UMIC (upper middle-income countries) 17% and HIC (high income countries) at 3% (see Table 5). Over a third of cases are from the following five countries: Nigeria (11%), India (7%), Uganda (7%), Tanzania (6%) and Kenya (5%). 

***Scale of operation*.** Large-scale fortification programmes accounted for 81% of the sample data (see Table 6), of which are predominantly maize or maize-related products (17%), wheat or wheat-related products (14%), rice (12%) and sweet potato (8%), see Table A2. 67% of large-scale programmes were considered successful (see Table 6). Furthermore, of the 81% of the programmes that distribute at a large-scale, 48% are mandatory, of which only 45% were successful. This figure excludes biofortification programmes which were all successful. Large scale voluntary fortification programmes showed a higher proportion of negative results (56%).

***Ownership structure.*** Out of the 103 cases, 56 described the programme’s ownership structure. 39% of the total cases were part of a multi-sector partnership, 9% led by the public sector, and 7% by the private sector. (see Table 7) However, from the cases which describe the programme’s ownership structure (56 cases), 71% were part of a multi-sector partnership, 16% led by the public sector, and 13% by the private sector. Results show fortification programmes operating in lower-income countries are more likely to be involved in cross-sector collaboration (see Table 8). 

***Regulatory environment.*** Biofortification programmes were categorised as ‘not stated’ in this study because currently there are no rules which govern the process of biofortification (however, these programmes are required to meet the national Food and Safety standards found in the country of operation). Therefore, out of the 61 regulated cases 77% were mandatory (51% successful), see Table 9. All milk and other flour (including cereal flour) programmes were mandatory. Moreover, 90% of sugar programmes, 95% of oil, 64% of wheat or wheat-related products and 31% of maize (including maize flour) were also operating in a mandatory environment (see Table A1). 

### 3.2. Scoring Criteria

**Figure 1:**
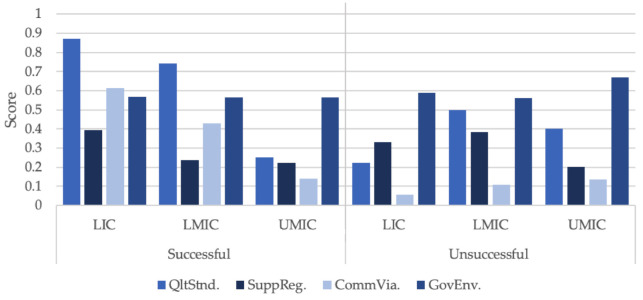
Mean score for LIC, LMIC and UMIC. LIC: low income countries; LIMIC: lower-and middle-income countries; UMIC: upper middle income countries; HIC: high income countries. Source: Author using data from Table A1.

Table 10 shows the mean and standard error for each of the scoring criteria. Figure 1 shows the mean score for each country type against *Target* (successful and unsuccessful). The main distinction between successful and unsuccessful cases can be seen by observing both *QltStnd* (*p* > 0.05) and *CommVia* (*p* > 0.05), whereby, successful cases scored higher than unsuccessful cases. For *QltStnd*, both LICs and LMICs in the successful category have a combined mean of 0.81 and 0.36 for unsuccessful cases. *CommVia* also show a similar score with both country groups, achieving a mean of 0.52 for successful cases and 0.08 for unsuccessful cases. A significant correlation was found between *QltStnd* and *Target* and between *CommVia* and Target (*p* < 0.05). No significant correlation was found between *SuppReg* and *Target* and between *GovEnv* and *Target*.

### 3.3. Developing the Typology

**Figure 2:**
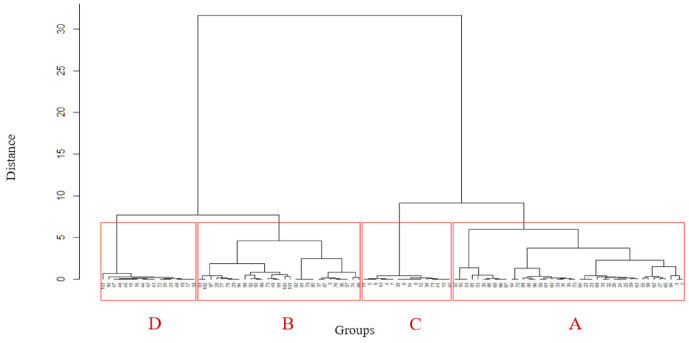
Cluster dendrogram used to derive the groups. Source: Author using data from Table A1.

Figure 2 shows a dendrogram resulting from a cluster analysis based on the five scoring criteria for each of the 103 cases in the data set. The R dist function was first to use to produce a distance matrix based on eculidean distance between the cases, and the hclust function then generated the dendrogram using Ward’s method. *X*-axis labels are the case numbers, and the *y*-axis scale is in Euclidean distance units, representing the criterion for a case to be included in a group. The tree was cut so as to produce the four groups, outlined in red.

Once the groups were establised, a non-metric multidimensional scaling (NMDS) model was developed to illustrate the distance between each case given the five scoring crtieia (*QltStnd*, *SuppReg*, *CommVia*, *GovEnv*, and *Target*). The NMDS model uses an ordination of replicates (cases), using measures shown as arrow labels. Each replicate is shown as a point, with colour coding the income group of the country involved. The chart has two orthogonal axes, with weightings for the measures indicated by the arrow points. Cases fall into four clear groups, whose properties are indicated in the legend. NMDS produces output resembling a principal components analysis, but is robust in the face of non-linear measurement scales [20], and deviations from normality, and correlations between the measures used. An iterative method is used to find stable solutions.

**Figure 3:**
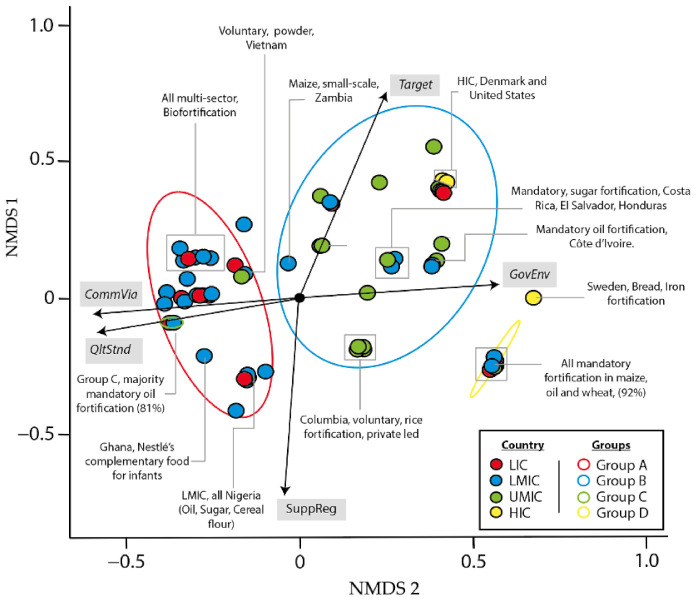
NMDS (Non-metric multidimensional scaling) using the total score for the 103 cases. Source: Author using data from Table A1.

**Figure 4:**
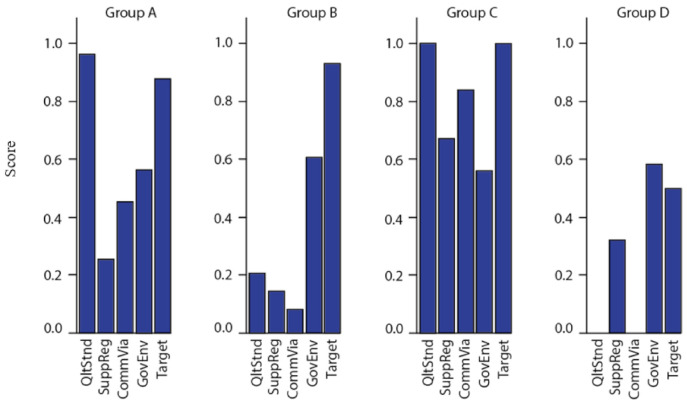
Mean score for each scording criteria Source: Author using data from Table A1.

Figure 3 shows each of the four groups, Group A (red): high quality standards identified with a good commercial environment but unregulated. Group B (blue): Low quality standards identified with some supportive regulations but predominantly unregulated. Group C (green): high quality standards identified with good supportive regulations and commercial environment. Group D (yellow): Low supportive regulations with a high rate of unsuccessful programmes. A significant correlation found between each of the four groups (A,B,C,D) and the following three criteria; country type, scale and fortification type (*p* < 0.05). Also, a significant correlation is found between each of the groups and *Target*. Figure 4 highlights the mean scores for each of the criteria by group.

Results from the analysis supported the construction of four main groups defined below: 

**Group A. high quality standards identified with a good commercial environment (40% of total cases).** In total, this group achieved a mean of 0.96 for *QltStnd*, 0.26 for *SuppReg,* 0.45 for *CommVia*, 0.56 for *GovEnv*, and *Target* 0.88. LICs accounted for 37% of the cases, LMIC’s (61%) and only a small proportio of cases were from UMIC’s (2%). This group achieved a high success rate of 76%, with LIC scoring 87%, LMIC 68% and UMIC 100%. Almost half of the programmes in this category are part of a multi-sector collaboration (49%), which are predominantly large scale (90%). 61% of these cases have not identified whether the fortification activity is mandatory or voluntary and are therefore considered unregulated. All cases marked as ‘non-stated’ for regulation were biofortified and are predominantly distributing at a large-scale (88%).

**Group B. Low quality standards identified with some supportive regulations (28% of total cases).** This group achieved a mean of 0.21 for *QltStnd*, 0.14 for *SuppReg*, 0.08 for *CommVia*, 0.60 for *GovEnv*, and *Target,* 0.93. The largest country group in this category are UMICs (48%) followed by LMIC (34%), LICs (10%) and HIC (7%). This group has also achieved a high success rate (86%), with all programmes in HIC, LIC and LMICs marked as successful and 71% of UMICs. 66% of cases did not state the programme’s model. Private-led programmes account for 17%, multi-sector 14% and public-led 3%. Both private-led and public-led programmes were found in UMICs. 69% of the programmes marked as ‘not stated’ for regulatory environment were biofortified. Mandatory cases account for 28% of which all were sucessful, whilst for voluntary cases (17%) only 20% were successful. Just over half of the cases are operating at a large-scale (59%) with only 7% as small-scale and 34% not stated. Large scale activities achieved 76% success, and both small and medium-scale distribution activities were marked as successful. 

**Group C. high quality standards identified with good supportive regulations and commercial environment (16% of total cases).** This group achieved a mean of 1 for *QltStnd,* 0.67 for *SuppReg*, 0.84 for *CommVia*, 0.56 for *GovEnv*, and *Target* at 1. LIC countries accounted for 69% of cases in this category, LMIC 25% and UMIC at 6%. In general, all cases in this category were successful and all were based on a cross-sector collabration, mandatory and distributing at a large-scale. The two vehicles found were oil (81%) and sugar (19%).

**Group D. Some supportive regulations with a high rate of unsuccessful programmes (17% of total cases).** This group achieved a mean of 0 for QltStnd, 0.32 for *SuppReg*, 0 for *CommVia*, 0.58 for *GovEnv*, and *Target* at 0.5. LMICs accounted for 47% of the cases, with LIC at 41%, UMICs at 6% and HICs at 6%. All cases in this group were reported as unsucessful with no discussion on the type of business. 88% of the cases were mandatory large-scale fortifcation programmes. 

### 3.4. Typology

**Figure 5:**
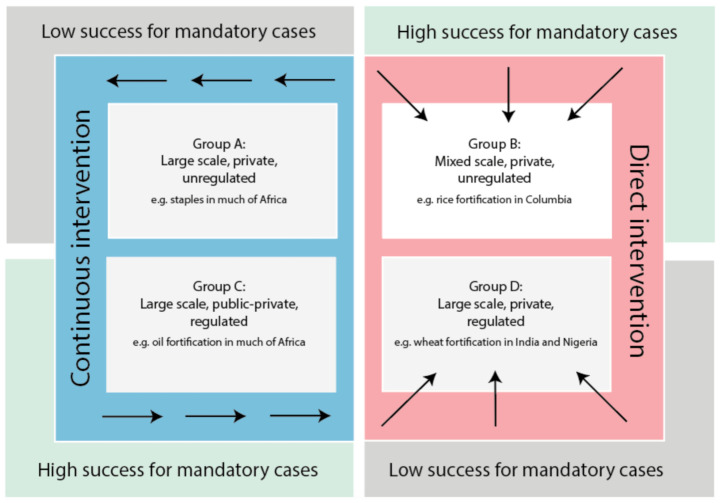
Typology Source: Author.

Based on the above analysis, the following typology is proposed (see Figure 5). The typology is based on the four groups identified in Section 3.3. It uses two factors, *QltStnd* and *CommVia* to determine the type of intervention (*continuous* or *direct intervention*) needed to improve programme success. Continuous intervention can be defined as the constant flow of public sector support in areas linked to distribution and access to knowledge and information. Direct intervention can be defined as the direct support necessary from the state to establish adequate policies and processes needed to improve the reach of fortified products. Please see Table 11 for a summary explanation. 

Groups A and C have cases with a high overall success and require constant support in areas linked to production and economies of scale. In essence, the continuity of support is necessary to strengthen current programmes and ensure firms achieve sustained growth. In Group B, whilst showing a high level of success, low levels of success for voluntary cases (80% unsuccessful) suggest a form of direct intervention is necessary to improve programme performance such as through quality monitoring infrastructure (e.g., standards/legilsation). In addition, cases within Group D show a low overall success and require direct intervention to overcome issues linked to programme structure and vehicle choice. (Please note whilst some cases may require both direct and continues intervention, this typology aims to illustrate the significance between both options and careful consideration should be given when developing and implementing policies to drive success.)

**Group A: Large scale, private, unregulated**. Successful private-led biofortification programmes in India and Pakistan [3] have been found to operate successfully in this group. Other activities led by the public-private sector under voluntary regulations have positively impacted the commercial environment [24]. Centralised production activities for staples (such as beans, rice, maize and wheat) improved quality assurance, especially in LICs and LMICs, where much of the output is exported. Good forms of monitoring/quality assurance are also found were supportive regulations are available [29]. Large-scale biofortification initiatives have a strong multi-sector partnership [3,30]. Such programmes require further investment in planting material (e.g., seed multiplication ability) and agronomic training to improve scale.It has also been suggested that for biofortification to be successful this requires breeding of varieties with high nutrient density accompanied by high yields that lead to better returns for farmers. Moreover, the micronutrient status of those consuming the biofortified varieties also needs to show improvement which requires appropriate retention during processing/cooking and which thereby ensures nutrients are ‘sufficiently bioavailable’. Lastly, scale needs to be achieved both in terms of adoption by farmers and consumption by those suffering from micronutrient malnutrition [31].

**Group B: Mixed scale, private, unregulated.** The need for state intervention to improve industry standards, choice of delivery channels, increase demand and protect commercial activities is necessary to improve programmes’ success. Good consumer knowledge of fortified foods is found in this group, especially among the upper-income countries. However, such knowledge does not always lead to an increase in consumption (as seen in the case of *Arroz Roa*, Columbia [27]). Low demand for commercially fortified products could be associated with higher cost when compared to other non-fortified products [6,27]. A need for either the state or the commercial sector to absorb the additional costs is necessary to ensure these products are competitively priced.

**Group C: Large scale, public-private, regulated**. Much of the activities found in this category is driven by collaborative effort between government, non-profit organisations and the private sector. The extent of such partnerships helped solve issues linked to resources, knowledge gap and market reach [26]. Successful implementation of mandatory policies helped create industry standards for large-scale fortified condiments such as oil and salt which enabled such products to reach all critical segments of the population.

**Group D: Large scale, private, regulated.** Though mandatory fortification exists, poor planning and implementation of large-scale production activities have limited the success of fortified vehicles such as maize and wheat [28]. Poorly designed regulations, lack of market control and monitoring activities created a vacuum that undermines the values associated with food fortification. A possible solution would be to centralise production activities to increase control. Once the right monitoring tools, training and knowledge for fortified foods have been established, both non-profit and commercial firms could leverage state resources to increase the reach of fortified products. 

To increase the success of fortification programmes and reduce micronutrient deficiency, either one of the following two actions is necessary. *Direct intervention* in markets where a lack of consumer knowledge, inadequate processing facilities, low to no regulatory/standards, poor distribution systems is found. *Continuous intervention* is required in markets that have shown a high level of success and have implemented clear monitoring systems. Such intervention is mainly focused on increasing scale, either vertically or horizontally along the value chain. A continuation in both state and donor support is necessary to increase fortified food’s commercial success. 

## 4. Discussion

The results have shown that it is possible to classify/group fortification initiatives by various attributes and reported success. Interestingly, the biofortification initiatives have all shown positive results, with the largest group of cases seen in lower-income countries (LIC 44% and LMIC 47%) of which 84% claim to be distributing at large scale. Complementary food showed a 67% success rate and fortification 53%.

Importantly, the findings indicate that where weaker forms of governance exist, strong multi-sector partnerships are required and associated with high rates of reported success. For example, in group A among LICs (80% that had established multi-sector partnerships) the vast majority reported a high-level of success (92%). Large scale fortification initiatives also showed a high success rate (87% of the cases that reported success in group A were large scale fortification initiatives compared to medium and small-scale initiatives). For example, a programme launched in Tanzania found it extremely difficult to build capacity for small firms due to the lack of supportive environment and regulatory framework [25]. Low success for mandatory programmes found in this group led to a high percentage of unsuccessful large-scale programmes. While it is correct to state that many large-scale fortification initiatives are often accompanied by mandatory legislation and that mandatory fortification (in LMIC contexts) is more likely to contribute to success in terms of providing a sustained source of fortified foods to the populace [32], this has not been the case for this group. Both the choice of food vehicle and lack of compliance with national standards [32] affected the commercial viability of mandatory programmes. Mkambula et al., (2020) identified 84 countries as ‘good candidates’ for new large-scale food fortification (LSFF) programs; among the criteria for selection include countries where centralised production is possible and where no legislation or voluntary legislation has been enacted [32].

Alongside this, barriers to success include political instability, political buy-in and lack of incentives (e.g., to bring industry onboard to fortify according to national standards) [32]. Others have noted that National Fortification Alliances (NFAs) are critical to supporting fortification initiatives especially with regards to contributing to oversight and guidance on how to improve the respective initiatives [26]. Thus, it is clear that in the category where governance metrics may be lower and/or supportive regulations; multi-sector partnerships with national level support can contribute to success. Other examples have been found where national and regional fortification alliances have been formed and stakeholders have supported industrial evaluations and engaged the private-sector [2]. 

One suggestion for combating difficulties with implementation is the use of third parties as complementary to regulatory inspectors. For example, consumer groups can play a role in identifying non-compliant brands/producers. This will reduce the burden on the inspectors and will allow them to then inspect those that have been identified by consumer groups [32]. There are some cases of successful private brands, such as, Nestlé’s range of complementary food for infants targeted at families with large disposable income in West Africa (Group A). By differentiating their product and positioning the brand as a ‘premium’ product, the company has been able to penetrate numerous markets in LMICS [33] though not without controversy. With respect to biofortifcation initiatives, whilst there has been success with respect to overall farmers willingness to produce/consume lack of planting material, sharing of information across farmer networks and unstable markets are some of the reasons cited for limited impact [34].

Among the contributing factors relating to reported success were strong monitoring capabilities and high levels of consumer awareness. Hoogendoorn et al. [2] has identified that ‘key success factors and preconditions’ for large scale fortification include tracking/reporting of the overall coverage of the population using the fortified foods and product quality safety monitoring. Furthermore, this also supports the groupings proposed by Timmer [35] that hypothesised with reference to large scale universal salt iodisation (USI) that a number of country groupings existed depending on their coverage, infrastructure and regulation etc. One particular group, for example were described as countries that have “scaled-up programmes where optimal coverage exists” but where there is a need to maintain oversight and ensure that disadvantaged/marginalised populations are reached. In contrast, another group identified limited coverage but consumer awareness, quality assurance and the improved capacity of producers (with appropriate policy/regulation support including incentives) were needed to reach scale (more akin to cases in group B). Examples of successful biofortification initiatives also involve strong public-private partnerships. For instance, in Bangladesh, SeedNet partnered with HarvestPlus a non-profit organisation that supports private firms’ entry new markets by subsidising the price for seeds sold directly to consumers [3]. NGOs and local states then supported the distribution of seed packs to local farmers [3]. Similarly, access to public healthcare systems is valuable for firms positioning their products in the complementary food market. This is seen in Vietnam with the Bibomix, a micronutrient powder given to children under 5 years of age [29]. However, it should be noted that strong state support is needed. An example of this is seen in Zambia in relation to a biofortification initiative. The coverage for the programme reached 75% when a subsidy was attached, but once subsidies were removed coverage dropped to about 33% [36] suggesting that ongoing government support for such programmes is necessary. Most activities are led by collaboration between public and private sectors. Opportunities to scale are supported by ‘interventionist’ policies such as the Farmer Input Support Program (FISP) in Zambia. The programme provides at least 50% subsidy for Maize seeds to vulnerable farmers [3]. Thus it is clear where there is a higher awareness of the benefits of fortified food mainly driven by strong social marketing efforts by the government suppliers in these markets are also able to charge a premium for their products [24]. There are challenges to successful outcomes of fortification initiatives. Caution should be raised regarding the sustainability and scale of these initiatives despite reported success. For example, many national fortification initiatives often struggle to reach very remote communities or scale due to inadequate funding mechanisms [37]. For biofortification initiatives, cases of success were reported, for example, in Rwanda where access to extension and informal dissemination through social networks were found to be key drivers of success. For example, it was found for iron-biofortified beans that proximity to planting material increases the rate of adoption and delivery of larger quantities to seed multipliers at the village level reduces ‘disadoption’ [30] (in group A). However, it should be noted that such programmes come with a high level of subsidy (financial or non-financial support). This is due to the lack of infrastructure and delivery systems needed to maximize the rate of coverage and returns (a comparison between Africa and Asia as mentioned by [36]).

In Group B, of the 86% that reported success (28% were mandatory cases), 80% were from LICs/LMICS combined. Where success has been found this is largely due to centralized production with mandatory legislation (which is easier to monitor and restricted to staples such as oil [26]). In Côte d’Ivoire, the government introduced new quality control devices, such as the iCheck CHROMA, which allowed for more rapid analysis and despite the precarious political situation this curbed the amount of unfortified oil flooding the market through porous borders in the North of the country [2]. Interestingly, where voluntary fortification was successful this enabled competition on quality and consumers were also well informed regarding the value of fortified food (high level of consumer awareness). Most upper-income countries in Latin America fit into this category (Brazil and Colombia). They have extensive experience in fortifying staple foods such as maize and wheat [38]. Food fortification activities are often a joint effort between public, private and the civil society and staple foods such as maize, wheat and rice. (See Table A2). Of particular note, however, is that ‘success’ is not restricted to higher income countries and LMICs accounted for many of the successful cases in this group. 

The main factors leading to ‘failure’ or a lack of success being reported relates primarily to poor implementation. A number of cases in this group were based on a wide-ranging review of eight countries (with mandatory fortification strategies) that highlighted a lack of impact. This is due in part to poor coverage, program design failures and lack of monitoring/evaluation, and learning [28]. For example, the main reasons for the lack of success were poor choice of food vehicle (i.e., bulk of the vehicle was not fortifiable or a staple), program design failures (including inability to include largescale producers/centralisation of production etc.) challenges with compliance/enforcement of fortification and failure to reach vulnerable groups (either because they do no consume the food vehicle or because of access/affordability) [28]. Similarly, other research has also highlighted numerous countries with mandatory programs in place that are also having difficulty with ‘effective implementation’ [32]. A recent assessment by the Food Fortification Initiative (FFI) in 2015 highlighted that of 84 countries that had mandatory legislation at the time for cereals (such as wheat flour, maize and/or rice) none had put in place appropriate monitoring tools [2]. It has been well documented that an important contributor to success of fortification initiatives are standards and national level agents that are able to enforce necessary regulations [2]. Lalani et al. [5] also showed in a study of stakeholders involved in industrial fortification from 35 countries that standards, large-scale production and in-factory testing were among the key factors associated with success/coverage of the target market in LMICs.

Group C has the highest reported success rate. It is clear that good quality standards and supportive regulations are associated with a high level of success for all types of fortification initiatives in this group. For example, the vegetable oil fortification programmes in Senegal and Mali a mandatory fortification decree for vegetable oil is in place and more importantly have enabled the enforcement of strong quality assurance protocols with the support of industry and the government. In Senegal, the programme was established within the Prime Minister’s Office with industry participation secured at the beginning of the project (i.e., industry were also involved with developing quality assurance/control protocols) and other government ministries also strongly supported the initiative by procurement of equipment which helped to offset initial costs [2]. 

The final group (group D) has a higher supportive regulations score (compared to group A and group B) with a high rate of unsuccessful programmes. This mostly consist of LICs and LMICs (88%) and are mainly large-scale (93%). The main contributing factors include poor implementation of mandatory policies, low consumer knowledge of food fortification, poor access to markets and an absence of product innovation [38]. Much of the failure is seen in fortification programmes linked to wheat related products in Nigeria, Tanzania and Uganda. 

## 5. Conclusions

Through analysis of a comprehensive literature review (published in this journal) of reported business model success and failures with respect to fortification and biofortification initiatives [4], we posit that it is possible to catergorise initiatives into four broad typology groups. We recognize that our approach has some deficiencies. For example, there is selection bias in the paper reviewed because success is more often reported than failure. Also, the method is, by nature, reductive, but we would argue that typologies are themselves prone to this criticism. We also recognize that, by including both fortification and biofortification in this analysis, comparison and therefore clustering of results is less sound than if these had been separated out. Notwithstanding, we believe that emerging typology adds to the discourse because it goes beyond a discussion on scale alone (i.e., ‘industrial’ vs. ‘small-scale’) and recognizes that both fortification and biofortification can be driven by commercial factors.

The implication of this new typology is that several factors need further consideration in designing future food fortification initiatives. These include:

- Mandatory regulation without quality infrastructure, leads to disappointing results (e.g., Group A—high quality standards identified with a good commercial environment but unregulated);
- Public-private partnership seems more successful in less structured low-income economies where value chains are highly dispersed and have many actors/actions but the vehicles were processed in a more centralized way, such as vegetable oil and sugar (e.g., Group C—high quality standards identified with good supportive regulations and commercial environment.);
- More coherent value chains find it easier to self-regulate in higher income economies and are not as impacted by scale as complex or dispersed value chains (Group B—Low quality standards identified with some supportive regulations but predominantly unregulated); and,
- Scale is not a success factor where other policies undermine fortification initiatives (particularly domestic regulation, market intervention by government and absence of quality infrastructure). i.e., Group D—(low supportive regulations with a high rate of unsuccessful programmes).

We conclude that analysing types of fortification efforts is useful for understanding what works and what does not work in order to improve the health of vulnerable communities. Future research might build more depth into this initial typology and question how policy interventions and background economic conditions might facilitate movement from one type to another or increase the success rate in specific types that seem to be under-performing.

## Figures and Tables

**Table 1:** Five steps to developing the typology.

Steps	Data	Thematic/Statistical Analysis	Results
Step 1. Literature search	Key word search found 106 articles, of which 34 were relevant for this study	ScienceDirect, Scopus, Google Scholar and Web of Science	34 articles. (Full list can be found in Table A1)
Step 2. Identifying cases	The articles were loaded onto NVivo to be analysed.	Information from each article were grouped into 17 different notes with each note representing a particular set of information. See Table 2 for details	17 nodes. See Table 2.
Stage 3. Building the database	Information from NVivo were used to develop a master spreadsheet. Each case logged represented one fortificant type (e.g., Iron, Zinc, Vitamin A etc.) and one method of fortification (e.g., spraying, coating etc.).	Both VLOOKUP/and Pivot Tables were used to summaries the data	955 cases were identified from 79 countries, of which 36 types of food vehicles were used, with minerals accounting for 34% and vitamins for 28%.
Stage 4. Statistical analysis	List of cases reduced from 955 to 263. This included only cases that have shown either successful or unsuccessful results. Cases that were associated with the same product were then grouped to be presented as one case. Cases with no country association were also removed from the final list. This helped narrowed the list from 263 to 103		103 cases from 41 countries, of which 16 types of food vehicles were used, with minerals accounting for 25% and vitamins for 60%.
Stage 5. developing the typology and further analysis	A set of four scoring criteria were developed using the data from Excel: *CommVia, SuppReg, QltStnd* and *Target.* A fifth criteria, *GovEnv,* was developed using data from The Heritage Foundation Index, Governance environment (ref)	Cluster analysis based on the scoring criteria was used to produce a dendrogram to define the groups. A non-metric multidimensional scaling (NMDS) was used to illustrate the distance between cases. A correlation test found significant correlation between *CommVia* and *Target* and between *QltStnd* to and *Target*. No correlation found between *SuppReg* and *Target,* also between *GovReg* and *Target.*	Four business model groupings identified: 1. Large-scale private, unregulated. 2. Mixed-Scale, private, unregulated. 3. Large-scale, public-private, regulated. 4. Large-scale, private, regulated.

**Table 2:** Summary analysis of relevant dimensions used in NVivo.

Initial Dimension of Food Fortification Business (Lalani et al., 2019)	Characteristic of Dimension
(1) Fortification	Mode of fortification (fortification, biofortification or supplementation foods?)
(2) Fortificant	The type of fortificant used for each food vehicle e.g., vitamins or mineral.
(3) Food vehicle	Choice of food(s) used to for the fortification type. For instance, commodity, food product (weaning, sprinkles, bar, yoghurt etc), crop
(4) Technology	The type of technology (or special methods) used for the fortification process e.g., processing, spraying, coating, pre-mix etc.
(5) Regulation	The type of legislations found i.e., mandatory or voluntary inclusion
(6) Standards	Types of control mechanisms used to enforce regulations. It covers monitoring tools, quality checks and also covers issues of commitment, compliance and willingness to ensure the fortification process/output is delivered.
(1) Programme success	Based on the authors definition of success and/or whether the programme reached target.
(2) Geographical coverage	Supra-national, national, regional, district, group/cooperative
(3) Countries/Region	The country of operation.
(4) Country type	Economic status of target country e.g., high-income country (HIC), upper-middle income country (UMIC) etc.
(5) Firm type and size	Information on firm size, for instance, small and medium sized firm (SME) or large firm. * If this is not stated but a firm name is provided, we estimated the size based on the volume of operation, capacity and market reach.
(6) Business model	Types of business model e.g., public led, private led and multi-sector partnerships
(7) Target group	Target customers/consumers and their characteristics e.g., whole population, specific vulnerable groups, age, gender, health status etc.
(8) Resources	Type of recourses used/needed to deliver the fortification programme e.g., financial support for small scale firms, data/information for biofortification programmes. Other resources include distribution networks such as national healthcare systems. Technology, machinery and material can also be considered as a resource which includes milling equipment, hammermills, blending mechanism
(9) Economics/costs	This includes any discussion on the cost of running the programme including margins, initial capital, cost of consumption (price) and production.
(10) Competition	Level/type competition between firms
(11) Marketing efforts	Branding and communicating the value of fortification programme

**Table 3:** Categories and description of fortificant type, food vehicle and country found for the 955 cases.

Categories	Description
Fortificant type	*Minerals:* Calcium, Copper, Iodine, Iron, Selenium, Zinc *Vitamins*: Provitamin A, Vitamin A, Vitamin B, Vitamin C, Vitamin D, Vitamin E, Vitamin K1.
Food vehicle used for fortification (*n* = 36)	Banana, Beans, Biscuits, Bread, Cakes, Canola, Capsule, Cassava, Chickpea, Corn, Cowpea, Edible oil, Flour (Cereal, Maize, Wheat), Lentil, Maize (and other related products such Orange Maize and Maize meal), Margarine, Milk, Monosodium glutamate, Noodles, Oil, Pasta, Pastries, Pearl Millet, Potato (and Sweet potato, Powder, Pumpkin, Rice (Regular, Golden and Ultra), Salt, Sorghum, Soybeans, Sprinkles, Sugar, Tomato, Vanaspati, Wheat (and other related products such as Wheat buns and Wheat grain), Yogurt.
Countries (*n* = 79)	Afghanistan, Angola, Argentina, Australia, Bangladesh, Benin, Bolivia, Brazil, Burkina Faso, Burundi, Cambodia, Cameroon, Canada, Chad, Chile, China, Colombia, Costa Rica, Cote d’Ivoire, Democratic Republic of Congo, Denmark, Dominican Republic, Ecuador, Egypt, El Salvador, Eritrea, Ethiopia, Gambia, Ghana, Guatemala, Guinea, Guinea-Bissau, Haiti, Honduras, India, Indonesia, Ireland, Kenya, Liberia, Madagascar, Maharashtra, Malawi, Malaysia, Mali, Mexico, Morocco, Mozambique, Myanmar, Nepal, Nicaragua, Niger, Nigeria, Oman, Pakistan, Panama, Papua New Guinea, Paraguay, Peru, Philippines, Rwanda, Senegal, Sierra Leone, South Africa, Sri Lanka, Sudan, Sweden, Switzerland, Tanzania, Thailand, Togo, Turkey, Uganda, United States, Uzbekistan, Venezuela, Vietnam, Yemen, Zambia, Zimbabwe.

**Table 4:** List of categories used to develop the typology and their score.

Final Index/Indicator	Scoring Objective	Categories Used from Table 2 to form Final Index/Indicators	Final Scoring Used for PCA/Scoring Explained
(1) Quality and standards (*QltStnd*)	To identify whether national micronutrient guidelines were stated and/or the quality of the fortified vehicle is in line with consumer expectation (based on taste and habit).	Standards identified	Score 0.5
		Quality identified	Score 0.5
(2) Supportive regulations/legislations/policies (*SuppReg*)	To identify the types of supportive regulations found in each case. Different scores were given to mandatory and voluntary fortification cases	Regulation of business space	0.25 if voluntary
			0.5 if mandatory
		Government support	0.5 if government support (e.g., subsides) provided
			0.5 if other government support provided
(3) Commercial viability (*CommVia*)	To identify the structure of the programme from ownership (collaboration between different sectors and firm size) and supply chain integration (i.e., vertical or horizontal integration)	Business/programme model	0.25 if led by multiple sectors
			0.17 if led by either the privet or public sector
		Collaboration (between different sized firms?)	0.25 if collaborated with different size firms
		Vertical integration	0. 17 if vertical integrated
		Horizontal integration	0. 17 if horizontally integration
		Integration with public systems	0.17 if integrated with public distribution systems
(4) Target reached (*Target*)	Identify if the programme showed positive results e.g., high uptake in fortification products, reduce the Disability-Adjusted Life Year (DALY) fatalities, successful distribution etc.	Programme success	1 if successful
			0.5 if not successful
(5) Governing Environment (*GovEnv*)	Information was obtained from the Index of Economic Freedom to represent case country profiles [14].	Scores range from 0 (low) to 1 (high)	

**Table 5:** Results for each country type.

Country type	*n*	Results
LIC	36	Biofortification (*n* = 16), all successful. Fortification (*n* = 20), 11 successful and 9 unsuccessful.
LMIC	47	Biofortification (*n* = 17), all successful. Complementary food (*n* = 3), 2 successful, and 1 unsuccessful. Fortification (*n* = 27), 12 successful 15 unsuccessful.
UMIC	17	Biofortification (*n* = 2), all successful. Fortification (*n* = 15), 10 successful 5 unsuccessful.
HIC	3	Biofortification (*n* = 1), all successful. Fortification (*n* = 2), 1 successful 1 unsuccessful.

LIC: low income countries; LIMIC: lower-and middle-income countries; UMIC: upper middle income countries; HIC: high income countries. Source: Author using data from Table A1.

**Table 6:** Results for scale and programme type.

Scale	*n*	Results
Large-scale	83	Biofortification (*n* = 29), all successful. Complementary food (*n* = 1), successful. Fortification (*n* = 53), 26 successful 27 unsuccessful.
Medium-scale	2	All Complementary food, 1 successfully and 1 unsuccessful.
Small-scale	4	Biofortification (*n* = 3), all successful. Fortification (*n* = 1), unsuccessful.
Not stated	14	Biofortification (*n* = 4), all successful. Fortification (*n* = 10), 8 successful 2 unsuccessful.

Source: Author using data from Table A1.

**Table 7:** Results for business model and programme type.

Ownership	*n*	Results for Each Programme Type
*Multi-sector*	40	Biofortification (*n* = 17), all successful. Complementary food (*n* = 1), all unsuccessful. Fortification (*n* = 22), 21 successful 1 unsuccessful.
Private sector	7	Biofortification (*n* = 2), all successful. Fortification (*n* = 5), 1 successful 4 unsuccessful.
Public-sector	9	Biofortification (*n* = 3), all successful. Complementary food (*n* = 2), all successful. Fortification (*n* = 4), 1 successful 3 unsuccessful.
Not stated	47	Biofortification (*n* = 14), all successful. Fortification (*n* = 33), 11 successful 22 unsuccessful.

Source: Author using data from Table A1.

**Table 8:** Results for business model and country type.

Ownership	*n*	Results for Each Programme Type
Multi-sector	40	LIC (*n* = 23), 22 successful, 1 unsuccessful. LMIC (*n* = 14), 13 successful, 1 unsuccessful. UMIC (*n* = 3), all successful.
Private sector	7	LMIC (*n* = 2), all successful. UMIC (*n* = 5), 1 successful, 4 unsuccessful
Public-sector	9	LIC (*n* = 1), all successful. LMIC (*n* = 7), 4 successful, 3 unsuccessful. UMIC (*n* = 1), all successful.
Not stated	47	LIC (n = 12), 4 successful, 8 unsuccessful. LMIC (*n* = 24), 12 successful, 12 unsuccessful. UMIC (*n* = 8), 7 successful, 1 unsuccessful. HIC (*n* = 3), 2 successful, 1 unsuccessful.

Source: Author using data from Table A1.

**Table 9:** Success of fortification/biofortification initiatives byregulation type.

Regulations	*n*	Results
Mandatory	47	◦ All Fortification, 24 successful 23 unsuccessful.
Mandatory (partial)	1	◦ Fortification and successful.
Voluntary	13	◦ Complementary food (*n* = 3), 2 successful 1 unsuccessful. ◦ Fortification (*n* = 10), 4 successful 6 unsuccessful.
Not stated	42	◦ Biofortification (*n* = 36), all successful. ◦ Fortification (*n* = 6), 5 successful, 1 unsuccessful.

Source: Author using data from Table A1.

**Table 10:** Mean and standard error for each scoring criteria.

Criteria	Mean Score	Standard Error
Quality and Standards *(QltStnd)*	0.59	0.04
Supportive Regulations *(SuppReg)*	0.30	0.02
Commercial Viability *(CommVia)*	0.33	0.30
Government Environment *(GovEnv)*	0.58	0.01
Target	0.85	0.02

Source: Author using data from Table A1.

**Table 11:** Explaintion of challenges which require continuous intervention and direct intervention.

Intervention Type	Example Challenge	Explaination
**Continuous intervnetion**	Egypt, bread fortification (Group A)	In [24] identified the need for continuous support (in subsidy) under the national Food Subsidy programme to ensure quality products are continually distributed to the wider population.
	Tanzania, salt fortification (Group A)	In [25] identified the need for further support in improving testing and training necessary to achieve a homogeneous concentration of iodine in salt.
	Senegal and Mali oil fortification (Group C)	In [26] Identified the need for continued public-private collaboration in areas linked to budgeting and capacity building.
**Direct intervnetion**	Columbia, Rice fortification (Group B)	In [27] identified two challenges that require direct intervention from the government (1) legislation which makes rice fortification mandatory, and (2) the need for price controls to ensure products are affordable and accessible to the wider population
	Nigeria, Uganda and Tanzania wheat fortification (Group D)	In [28] Identified the need for direct intervention to improve the choice of fortified vehicles and programme design.

## Data Availability

The data is available upon request.

## Appendix A

**Table A1:** List of articles used for the typology.

No.	Reference	Used in Stage 3	Referenced in the Typology
1	Aaron, G.J., et al., *Assessing Program Coverage of Two Approaches to Distributing a Complementary Feeding Supplement to Infants and Young Children in Ghana.* PLoS One, 2016. **11**(10): p. e0162462.	✓	✓
2	Aaron, G.J., et al., *Coverage of Large-Scale Food Fortification of Edible Oil, Wheat Flour, and Maize Flour Varies Greatly by Vehicle and Country but Is Consistently Lower among the Most Vulnerable: Results from Coverage Surveys in 8 Countries.* The Journal of Nutrition, 2017. **147**(5): p. 984S-994S.	✓	✓
3	Assey, V.D., et al., *Improved salt iodation methods for small-scale salt producers in low-resource settings in Tanzania.* 2009. **9**(1): p. 187.	✓	✓
4	Baltussen, R., C. Knai, and M. Sharan, *Iron fortification and iron supplementation are cost-effective interventions to reduce iron deficiency in four subregions of the world.* The Journal of nutrition, 2004. **134**(10): p. 2678–2684.	✓	X
5	Beinner, M.A., et al., *Iron-Fortified Rice Is As Efficacious As Supplemental Iron Drops in Infants and Young Children.* The Journal of Nutrition, 2010. **140**(1): p. 49–53.	✓	✓
6	Bouis, H.E. and A. Saltzman, *Improving nutrition through biofortification: A review of evidence from HarvestPlus, 2003 through 2016.* Global Food Security, 2017. **12**: p. 49–58.	✓	✓
7	Chilimba, A.D.C., et al., *Agronomic biofortification of maize with selenium (Se) in Malawi.* Field Crops Research, 2012. **125**: p. 118–128.	✓	✓
8	Darnton-Hill, I. and R. Nalubola, *Fortification strategies to meet micronutrient needs: successes and failures.* Proceedings of the Nutrition Society, 2002. **61**(02): p. 231–241.	✓	✓
9	De Groote, H., et al., *The effectiveness of extension strategies for increasing the adoption of biofortified crops: the case of quality protein maize in East Africa.* Food Security, 2016. **8**(6): p. 1101–1121.	✓	✓
10	Elhakim, N., et al., *Fortifying baladi Bread in Egypt: Reaching More than 50 Million People through the Subsidy Program.* 2012. **33**(4 suppl3): p. S260-S271.	✓	✓
11	Fiedler, J.L. and B. Macdonald, *A Strategic Approach to the Unfinished Fortification Agenda: Feasibility, Costs, and Cost-Effectiveness Analysis of Fortification Programs in 48 Countries.* 2009. **30**(4): p. 283–316.	✓	X
12	Fiedler, J.L. and C. Puett, *Micronutrient program costs: Sources of variations and noncomparabilities.* Food and Nutrition Bulletin, 2015. **36**(1): p. 43–56.	✓	X
13	Fiedler, J.L., et al., *Maize flour fortification in Africa: markets, feasibility, coverage, and costs.* Annals of the New York Academy of Sciences, 2014. **1312**(1): p. 26–39.	✓	X
14	Forsman, C., et al., *Rice fortification: A comparative analysis in mandated settings.* Annals of the New York Academy of Sciences 2014. **1324**(1): p. 67–81.	✓	X
15	Garrett, G.S., C. Manus, and A. Bleuthner, *Chapter 11—The Importance of Public–Private Collaboration in Food Fortification Programs*, in *Food Fortification in a Globalized World*, M.G.V. Mannar and R.F. Hurrell, Editors. 2018, Academic Press. p. 113–120.	✓	✓
16	González, C., N. Johnson, and M. Qaim, *Consumer acceptance of second-generation GM Foods: The case of biofortified cassava in the North-east of Brazil.* Journal of Agricultural economics, 2009. **60**(3): p. 604–624.	✓	✓
17	Greiner, T., *Fortification of processed cereals should be mandatory.* Lancet, 2007. **369**: p. 1766–1768.	✓	✓
18	Gómez-Galera, S., et al., *Critical evaluation of strategies for mineral fortification of staple food crops.* Transgenic Research, 2010. **19**(2): p. 165–180.	✓	✓
19	Horton, S., *The Economics of Food Fortification.* The Journal of Nutrition, 2006. **136**(4): p. 1068–1071.	✓	X
20	Hotz, C., et al., *Efficacy of Iron-Fortified Ultra Rice in Improving the Iron Status of Women in Mexico.* 2008. **29**(2): p. 140–149.	✓	✓
21	Humphrey, J. and E. Robinson, *Markets for Nutrition: What Role for Business?* IDS Bulletin, 2015. **46**(3): p. 59–69.	✓	✓
22	Jenkins, M., et al., *Factors affecting farmers’ willingness and ability to adopt and retain vitamin A-rich varieties of orange-fleshed sweet potato in Mozambique.* Food Security, 2018. **10**(6): p. 1501–1519.	✓	✓
23	Kaput, J., et al., *Enabling nutrient security and sustainability through systems research.* Genes & nutrition, 2015. **10**(3): p. 12.	✓	X
24	Meenakshi, J., *Biofortification. Best practice paper: new advice from CC08*. 2009: Copenhagen Consensus Center.	✓	✓
25	Muange, E.N. and A. Oparinde, *Social Network Effects on Consumer Willingness to Pay for Biofortified Crops.* 2018.	✓	X
26	Nguyen, M., et al., *A Delivery Model for Home Fortification of Complementary Foods with Micronutrient Powders: Innovation in the Context of Vietnamese Health System Strengthening.* Nutrients, 2016. **8**(5): p. 259.	✓	✓
27	Hunter, D., et al., *Enabled or Disabled: is the environment right for Using Biodiversity to improve Nutrition?* Frontiers in nutrition, 2016. **3**: p. 14.	✓	X
28	Ogunmoyela, O.A., et al., *A Critical Evaluation of Survey Results of Vitamin A and Fe Levels in the Mandatory Fortified Food Vehicles and Some Selected Processed Foods in Nigeria.* 2013. **31**(2): p. 52–62.	✓	✓
29	Osendarp, S.J.M., et al., *Large-Scale Food Fortification and Biofortification in Low- and Middle-Income Countries: A Review of Programs, Trends, Challenges, and Evidence Gaps.* Food and Nutrition Bulletin, 2018. **39**(2): p. 315–331.	✓	✓
30	Smale, M., et al., *The Changing Structure of the Maize Seed Industry in Zambia: Prospects for Orange Maize.* 2015. **31**(1): p. 132–146.	✓	X
31	Stein, A.J., et al., *Plant breeding to control zinc deficiency in India: how cost-effective is biofortification?* Public health nutrition, 2007. **10**(5): p. 492–501.	✓	✓
32	Tsang, B.L., et al., *Public and Private Sector Dynamics in Scaling Up Rice Fortification.* Food and Nutrition Bulletin, 2016. **37**(3): p. 317–328.	✓	✓
33	Vaiknoras, K., et al., *Promoting rapid and sustained adoption of biofortified crops: What we learned from iron-biofortified bean delivery approaches in Rwanda.* Food Policy, 2019. **83**: p. 271–284.	✓	✓
34	Zimmerman, S., et al., *Mandatory policy: Most successful way to maximize fortification’s effect on vitamin and mineral deficiency.* Indian Journal of Community Health, 2014. **26**(Supp 2): p. 369–374.	✓	X

**Table A2:** Key characteristics for each case by group and scores (Please note complementary food here includes targeted fortification e.g., in relation to infants and/or home fortification such the use of micronutrient powders (MNPs) used with complementary foods or MNPs mixed with school meals (point-of-use fortification). In a few cases, business models that may be defined as community-led either had a strong public-sector/civil society component and have been included under either public-sector led/multi-sector partnerships for ease of interpretation).

Group	Food vehicle (Figures Represent the Total Average per Vehicle in the Group)	Fortification Type	Scale	Business Model	Country	Legislation
Group A	Beans 12% (*n* = 5)	All biofortification	All large-scale	Multi-sector 80% (*n* = 4) Public-led 20% (*n* = 1)	LIC 80% (*n* = 4) LMIC 20% (*n* = 1)	All not stated
	Cassava 7% (*n* = 3)	All biofortification	Small-scale 33% (*n* = 1) Large-scale 67% (*n* = 2)	Multi sector 33% (*n* = 1) Not stated 33% (*n* = 1) Public-led 33% (*n* = 1)	All LMIC	All not stated
	Maize (All maize products including maize flour) 22% (*n* = 9)	Biofortification 67% (*n* = 6) Fortification 33% (*n* = 3)	All large-scale	Multi sector 67% (*n* = 6) Not stated 33% (*n* = 3)	LIC 56% (*n* = 5) LMIC 44% (*n* = 4)	Mandatory 11% (*n* = 1) Not stated 67% (*n* = 6) Voluntary 22% (*n* = 2)
	Not stated (Cases where no vehicles were referenced) 2% (*n* = 1)	All complementary food	All large-scale	All public-led	All LIC	All voluntary
	Oil 7% (*n* = 3)	All fortification	All large-scale	Multi sector 33% (*n* = 1) Not stated 33% (*n* = 1) Public-led 33% (*n* = 1)	All LMIC	Mandatory 67% (*n* = 2) Voluntary 33% (*n* = 1)
	Other flour including cereal 5% (*n* = 2)	All fortification	All large-scale	Not stated 50% (*n* = 1) Public-led 50% (*n* = 1)	All LMIC	All mandatory
	Pear Millet 2% (*n* = 1)	All biofortification	All large-scale	All multi sector	All LMIC	All not stated
	Powder 5% (*n* = 2)	All complementary food	All medium-Scale	Multi sector 50% (*n* = 1) Public-led 50% (*n* = 1)	All LMIC	All voluntary legislation
	Rice 7% (*n* = 3)	Biofortification 67% (*n* = 2) Fortification 33% (*n* = 1)	Not stated 33% (*n* = 1) Large-scale 67% (*n* = 2)	Multi sector 33% (*n* = 1) Not stated 67% (*n* = 2)	LMIC 67% (*n* = 2) UMIC 33% (*n* = 1)	Not stated 67% (*n* = 2) Voluntary 33% (*n* = 1)
	Salt 1% (*n* = 1)	All fortification	All small-scale	All multi sector partnerships	All LIC	All mandatory
	Sugar 5% (*n* = 2)	All fortification	All large-scale	Not stated 50% (*n* = 1) Public-led 50% (*n* = 1)	All LMIC	All mandatory
	Sweet potato 10% (*n* = 4)	All biofortification	All large-scale	Multi sector 50% (*n* = 2) Not stated 25% (*n* = 1) Public-Led 25% (*n* = 1)	All LIC	All not stated
	Wheat (All wheat products including wheat flour and grain) 12% (*n* = 5)	Biofortification 80% (*n* = 4) Fortification 20% (*n* = 1)	Not stated 20% (*n* = 1) Large-scale 80% (*n* = 4)	Multi sector 40% (*n* = 2) Not stated 20% (*n* = 1) Private 40% (*n* = 2)	LIC 20% (*n* = 1) LMIC 80% (*n* = 4)	Mandatory (partial) 20% (*n* = 1) Not stated 80% (*n* = 4)
Group B	Beans 3% (*n* = 1)	All biofortification	All large-scale	All not stated	All LIC	All not stated
	Cassava 3% (*n* = 1)	All biofortification	All not stated	All multi-sector	All UMIC	All not stated
	Maize 14% (*n* = 4)	Biofortification 50% (*n* = 2) Fortification 50% (*n* = 2)	Small-scale 25% (*n* = 1) Medium-scale 25% (*n* = 1) Large-scale 50% (*n* = 2)	All not stated	LMIC 50% (*n* = 2) UMIC 50% (*n* = 2)	Mandatory 25% (*n* = 1) Not stated 75% (*n* = 3)
	Margarine 3% (*n* = 1)	All fortification	All large scale	All not stated	All HIC	All not stated
	Milk 3% (*n* = 1)	All fortification	All not stated	All public-led	All UMIC	All mandatory
	Oil 3% (*n* = 1)	All fortification	All large scale	All not stated	All LMIC	All mandatory
	Rice 38% (*n* = 11)	Biofortification 36% (*n* = 4) Fortification 64% (*n* = 7)	Small-scale 9% (*n* = 1) Not stated 18% (*n* = 2) Large-scale 73% (*n* = 8)	Not stated 55% (*n* = 6) Private-led 46% (*n* = 5)	HIC 9% (*n* = 1) LMIC 36% (*n* = 4) UMIC 55% (*n* = 6)	Not stated 55% (*n* = 6) Voluntary 45% (*n* = 5)
	Sugar 17% (*n* = 5)	All fortification	Not stated 80% (*n* = 4) Large-scale 20% (*n* = 1)	Multi-sector 60% (*n* = 3) Not stated 40% (*n* = 2)	LMIC 60% (*n* = 3) UMIC 40% (*n*-2)	Mandatory 80% (*n* = 4) Voluntary 20% (*n* = 5)
	Sweet potato 10% (*n* = 3)	All biofortification	All large scale	All not stated	LIC 67% (*n* = 67%) UMIC 33% (*n* = 1)	All not stated
	Wheat 3% (*n* = 1)	All fortification	All not stated	All not stated	All UMIC	All mandatory
Group C	Oil 81% (*n* = 13)	All fortification	All large-scale	All multi-sector	LIC 85% (*n* = 11) LMIC 15% (*n* = 15%)	All mandatory
	Sugar 19% (*n* = 3)	All fortification	All large-scale	All multi-sector	LMIC 67% (*n* = 2) UMIC 33% (*n* = 1)	All mandatory
Group D	Bread 6% (*n* = 1)	All fortification	All not stated	Not stated	All HIC	All not stated
	Maize 18% (*n* = 3)	All fortification	All large-scale	Not stated	LIC 67% (*n* = 2) LMIC 33% (*n* = 33)	All mandatory
	Oil 24% (*n* = 4)	All fortification	All large-scale	Not stated	LIC 50% (*n* = 1) LMIC 50% (*n* = 1)	All mandatory
	Salt 6% (*n* = 1)	All fortification	All not stated	Not stated	All LMIC	All voluntary
	Wheat 47% (*n* = 8)	All fortification	All large-scale	Not stated	LIC 38% (*n* = 3) LMIC 50% (*n* = 4) UMIC 13% (*n* = 1)	All mandatory
